# Information Usage and Compliance with Preventive Behaviors for COVID-19: A Longitudinal Study with Data from the JACSIS 2020/JASTIS 2021

**DOI:** 10.3390/healthcare10030521

**Published:** 2022-03-13

**Authors:** Taro Kusama, Sakura Kiuchi, Kenji Takeuchi, Takaaki Ikeda, Noriko Nakazawa, Anna Kinugawa, Ken Osaka, Takahiro Tabuchi

**Affiliations:** 1 Division for Regional Community Development, Liaison Center for Innovative Dentistry, Tohoku University Graduate School of Dentistry, Sendai 980-8575, Japan; kenji.takeuchi.c4@tohoku.ac.jp; 2 Department of International and Community Oral Health, Tohoku University Graduate School of Dentistry, Sendai 980-8575, Japan; sakura.kawamura.r2@dc.tohoku.ac.jp (S.K.); t.ikeda0110@gmail.com (T.I.); noriko.umehara.s6@dc.tohoku.ac.jp (N.N.); kinugawa.annna.r3@dc.tohoku.ac.jp (A.K.); ken.osaka.e5@tohoku.ac.jp (K.O.); 3 Department of Health Policy Science, Graduate School of Medical Science, Yamagata University, Yamagata 990-9585, Japan; 4 Cancer Control Center, Osaka International Cancer Institute, Osaka 541-8567, Japan; tabuchitak@gmail.com

**Keywords:** COVID-19, preventive behavior, media, longitudinal

## Abstract

The influence of different types of information sources on individual preventive behaviors remains unclear. We aimed to investigate the associations between individual information usage to obtain information about COVID-19 and compliance with preventive behaviors. This longitudinal study was based on an Internet survey conducted in August–September 2020 and February 2021. We used compliance with four preventive behaviors for COVID-19, “wearing a mask”, “ventilation”, “social distancing”, and “avoiding crowds” as outcome variables, and 20 types of information sources based on people or institutions (Medical worker, Government, etc.) and media (TV news, Twitter, etc.) as predictors. Absolute differences and 95% confidence intervals were estimated using generalized estimating equations adjusted for possible confounders. Among the 18,151 participants aged 20–79, the mean age was 51.7 (SD = 15.9) in 2020, and 51.3% were male. In total, compliance with “wearing a mask”, “ventilation”, “social distancing”, and “avoiding crowds” was seen in 86.2%, 46.9%, 45.4%, and 62.6% of individuals in 2020, and 89.3%, 38.2%, 47.2%, and 61.6% of individuals in 2021, respectively. In the multivariate analysis, “medical workers”, “professionals”, “the government”, “Twitter”, “news websites”, and “TV news” were positively associated with compliance with two or more preventive behaviors (*p* < 0.05). The type of information source may play an important role in providing information for COVID-19 prevention.

## 1. Introduction

The coronavirus disease 2019 (COVID-19) pandemic, caused by severe acute respiratory coronavirus 2 (SARS-CoV-2), is an ongoing public health threat [1]. Although vaccinations have been developed to prevent COVID-19 [2], non-pharmaceutical interventions to prevent infection are still required because of the delayed progression of vaccination uptake and the mutation of the pathogenicity of SARS-CoV-2 [3]. Previous studies have suggested the effectiveness of physical distancing, wearing a mask, and building ventilation in preventing COVID-19 [4,5], and many political sectors have emphasized compliance with these preventive behaviors [6].

Previous studies have suggested that exposure to specific information sources, such as the media, government, professionals, and other people, affects individual preventive behaviors such as vaccination uptake and behavioral prevention strategies [7]. Social media has recently emerged as a vehicle for strong social impacts and is commonly regarded as a source of misinformation, including for COVID-19 [8,9]. Exposure to social media may affect individual knowledge and risk perception for COVID-19 prevention. A previous study among young adults suggested that individual knowledge and risk perception were associated with engagement in preventive behaviors for COVID-19 [10]. Several studies have shown the associations between social media use and non-compliance with preventive behaviors for COVID-19; however, only a few studies have investigated the associations between preventive behaviors for COVID-19 and multiple information sources [11,12,13,14]. In addition, most previous studies were cross-sectional and did not consider longitudinal changes in compliance with preventive behaviors.

The present study aims to evaluate the association of the use of multiple information sources based on people or institutions and media to obtain the information about COVID-19 prevention with the compliance of four preventive behaviors, including “wearing a mask”, “ventilation”, “social distancing”, and “avoiding crowds” for COVID-19 over a period of two years

## 2. Materials and Methods

### 2.1. Study Design and Participants

The present study is a longitudinal study using the two-point panel data obtained from the Japan COVID-19 and Society Internet Survey (JACSIS) and the Japan Society and New Tobacco Internet Survey (JASTIS). The JACSIS aimed to evaluate the health conditions and social determinants of the COVID-19 pandemic in Japan. JASTIS aimed to evaluate the status of new tobacco products and their related factors in Japan. Both surveys were administered on internet questionnaires. JACSIS, the baseline survey in 2020, was distributed to 224,389 candidates registered as panelists at a Japanese internet research company (Rakuten Insight, Inc., Tokyo, Japan) from 25 August to 30 September 2020. The selected sample (*n* = 28,000) is representative of the sociodemographic distribution of the Japanese population (sex, age, and residential prefecture). In 2021, the JASTIS survey was distributed to the participants from the eighth to the twenty-sixth of February. The participants who provided unreliable responses (i.e., selected “all” in questions regarding current using drugs or chronic diseases) were excluded from the analysis. Participants below 20 years of age or who answered their educational attainment as “other” were also excluded.

### 2.2. Outcome Variables: Compliance with Preventive Behaviors for COVID-19

We used compliance with preventive behaviors for COVID-19 in the 2020 and 2021 surveys as the outcomes. We selected four preventive behaviors: “wearing a mask”, “ventilation”, “social distancing”, and “avoiding crowds”. These outcomes were the preventive behaviors mandated by the Japanese governments’ campaign called “3Cs”, and multiple media outlets distributed the message [15]. The campaign calls public attention to avoid “closed spaces”, “crowded places”, and “close-contact settings”. We selected the following item from the JACSIS and JASTIS surveys to extrapolate our outcome variable, “In the last month, how did you often take any of the following preventive measures?” The outcome variable was extrapolated from four responses assessing preventive measures behaviors, namely “I wore a mask in a public place”, “I opened the window to ventilate the room”, “I tried to keep a social distance (at least 2 m from people)”, and “I tried not to go to places where people were crowded”. The questionnaire items used an ordinal scale, and participants selected their responses from “always”, “sometimes”, “seldom”, and “never”. We defined those who answered “always” as having compliance with each of four preventive measures.

### 2.3. Predictors: Information Usage to Obtain Information about COVID-19

To evaluate information usage, we used the types of information sources based on people or institutions and media that individuals use to obtain information about COVID-19 as predictors. We selected the following item from the JACSIS and JASTIS surveys: “Did you get information about the new coronavirus infection from each of the following sources?” Participants were able to select the following 20 types of information sources: (1) family, (2) friends, (3) workplaces and schools, (4) medical workers, (5) celebrities, (6) professionals, (7) government, (8) academic institutions, (9) video sharing sites (YouTube, etc.), (10) LINE, (11) Twitter, (12) Facebook, (13) Instagram, (14) news websites, (15) newspapers, (16) magazines, (17) books, (18) TV news, (19) TV tabloid shows, and (20) radio. Items one to eight are people and institutions from which participants sourced information about COVID-19. Items nine to twenty are the media-based information sources, while nine to thirteen are “social network services (SNS)”. For each information source, participants responded “yes” or “no”. We also included the time point of the response (2020 or 2021) as a predictor.

### 2.4. Covariates

We selected several covariates based on the previous studies and clinical knowledge [11,16,17,18]. All covariates were measured in 2020. The covariates included in the analysis were sex (male or female), age (20–29, 30–39, 40–49, 50–59, 60–69, or 70–79), equivalent income by quartile (Q1: <22,500 USD, Q2: 22,500–31,800 USD, Q3: 31,800–47,500 USD, Q4: >47,500 USD (1USD ≈ 100 JPY)), educational attainment (junior high school or high school, vocational school or junior college, or university or graduate-school), family structure (living with others or living alone), and health literacy measured using the Communicative and Critical Health Literacy (CCHL) scale. The CCHL consists of five questions regarding health literacy, and the average of the responses on a five-point Likert scale (1–5) presents the respondents’ health literacy [19].

### 2.5. Statistical Analysis

We estimated the absolute differences in percentages and 95% confidence intervals (95% CIs) for each preventive measure based on the generalized estimating equations fitting the Gaussian distribution and identity link with a Huber–White sandwich estimator for standard errors [20]. The time-variant variables were compliance with each preventive measure and the time point of the response, and the time-invariant variables were information usage and all covariates. Two-point panel data were created for each participant in a long format. The same values were inserted for time-invariant variables; thus, two-row data for each participant were inserted, with the outcome variable measured at either baseline or follow-up. Further, we inserted predictors and covariates measured at baseline as baseline and follow-up values. We built two models. Model 1 included each predictor of information usage or time point, adjusting all covariates separately to evaluate the association of each predictor with preventive behavior conditional on the included covariate. Model 2 included all predictors related to the types of people- or institution-based information sources (items one to eight) or media-based information sources (items nine to twenty) separately, with time points and all covariates. Model 2 aimed to evaluate the association of each predictor with preventive behavior conditional on other predictors and covariates. To reduce selection bias, all statistical analyses employed the inverse probability weighting method using the propensity score estimated from the Comprehensive Survey of Living Conditions, a representative Japanese sociodemographic random sample [21]. We also checked the differences in baseline characteristics between the original population and the analyzed population using standardized differences [22]. A standardized difference > 0.1 indicates a non-negligible difference between them. We used STATA 16.1 for all analyses.

### 2.6. Ethical Issues

JACSIS in 2020 and JASTIS in 2021 followed the procedures approved by the Ethics Committee on Research of Human Subjects at the Research Ethics Committee of the Osaka International Cancer Institute (No.20084-2). Additionally, we followed the STROBE statement to report our observational study.

## 3. Results

**Figure 1:**
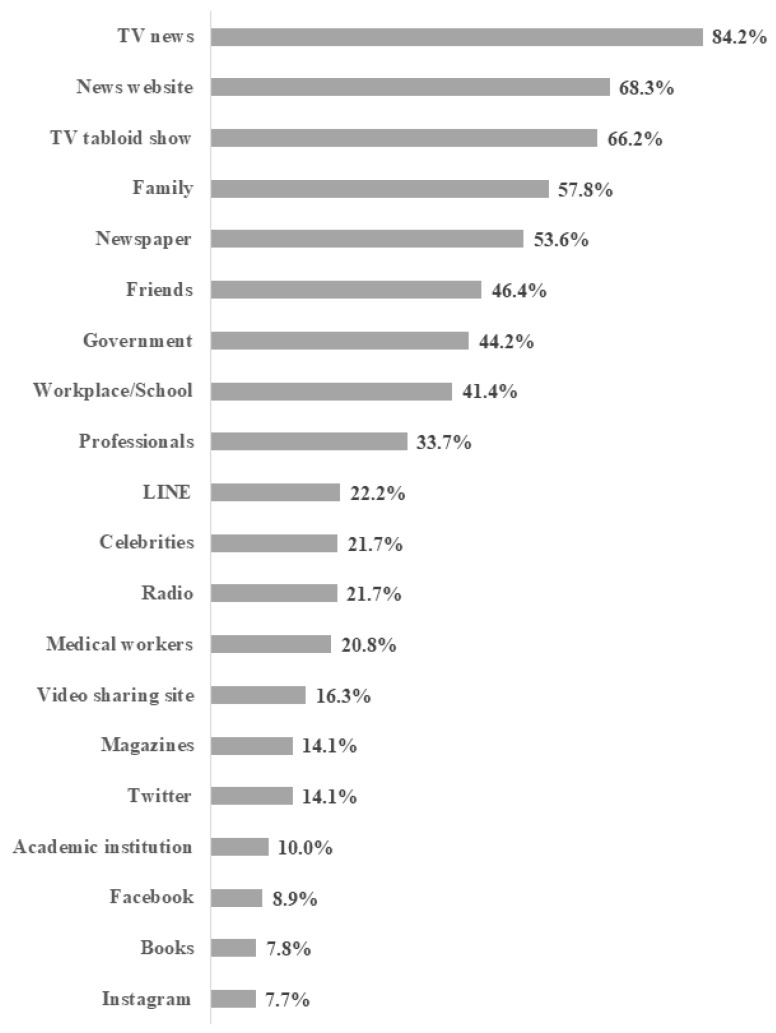
Weighted proportions of the use of information sources (*n* = 18,151).

The flowchart of participant inclusion is presented in Supplementary Figure S1. Initially, of the 28,000 participants who answered the JACSIS survey in 2020, 26,646 were aged 20 years or older and 24,208 were eligible at baseline. In the JASTIS 2021 survey, 20,061 (82.9%) participants responded. Finally, 18,151 participants were included in the analysis after excluding unreliable responses. We could not find a negligible difference in the distribution of baseline characteristics between the original and analyzed populations (Supplementary Table S1). The mean age of the participants was 51.7 (SD = 15.9) in 2020, and 51.3% were male. Table 1 shows the distribution of the participants’ characteristics. The proportions of those who had compliance with “wearing a mask”, “ventilation”, “social distancing”, and “avoiding crowds” were 86.2%, 46.9%, 45.4%, and 62.6% in 2020, and 89.3%, 38.2%, 47.2%, and 61.6% in 2021, respectively. The proportion of each predictor of information usage is presented in Table 1 (unweighted) and Figure 1 (weighted). The most used information source was “TV news” (84.2%) and the least used was “Instagram” (7.7%). Table 2 shows the proportions of those who complied with each preventive behavior based on information usage and covariates. The proportion of usage of each information source by sociodemographic and socioeconomic status is presented in Supplementary Table S2.

Table 3 shows the association of each predictor of information usage and compliance with each preventive behavior. In model 2, which included all predictors, most predictors were associated with significantly higher compliance with at least one preventive measure. Especially, for people- and institution-based information sources, “medical workers” (5.9% for ventilation (95% CI [1.2, 10.6] and 10.8% for social distancing (95% CI [6.3, 15.2])), “professionals” (5.7% for wearing a mask (95% CI [2.1, 9.3]) and 5.0% for ventilation (95% CI [0.1, 10.0])), and “government” (3.9% for wearing a mask (95% CI [1.3, 6.5]) and 6.7% for avoiding crowds (95% CI [3.4, 9.9])) were associated with significantly higher compliance with two of the four types of preventive behaviors. For media-based information sources, “Twitter” (3.8% for wearing a mask (95% CI [0.6,7.0]), 4.7% for ventilation (95% CI = 4.7 (95% CI [1.0, 8.3]), and 6.4% for social distancing (95% CI [3.0, 9.8])), “news website” (7.3% for wearing a mask (95% CI [5.1, 9.5]), 5.5% for ventilation (95% CI [1.5, 9.5]), and 5.5% for avoiding crowds (95% CI [2.1, 8.8]), and “TV news” (12.3% for wearing a mask (95% CI [8.2, 16.3]), 5.6% for avoiding crowds (95% CI [0.4, 10.7]) were associated with two or three of the four types of preventive behaviors. In contrast, “Celebrities” (−4.7% for avoiding crowds (95% CI [−9.0, −0.3])), “Instagram” (−18.9% for wearing a mask (95% CI [−28.8, −8.9])), “Facebook” (−6.8% for social distancing (95% CI [−12.2, −1.3])), and “newspaper” (−2.8% for ventilation (95% CI [−5.9, −0.3])) were negatively associated with compliance with at least one preventive behavior, respectively. Regarding changes due to time, compliance with ventilation decreased at follow-up by −9.3% (95% CI [−11.2, −7.5%]); meanwhile, compliance with social distancing increased by 3.0% (95% CI [1.2, 4.8]).

## 4. Discussion

The present study evaluated the association between the various types of information usage and compliance with four preventive behaviors: “wearing a mask”, “ventilation”, “social distancing”, and “avoiding crowds”, and how their compliance changed over time. The results of the present study showed that most information sources were positively associated with compliance with at least one preventive behavior. Information sources including “medical workers”, “professionals”, “government”, “Twitter”, “news website”, and “TV news” were especially positively associated with compliance with two or more preventive behaviors for COVID-19.

Previous studies have also suggested positive or negative associations between the information sources and compliance with preventive behaviors. Although the classification of the information sources in the present study was defined differently than in previous studies, the findings of the present study corroborated previous studies that stated that the use of information sources is associated with preventive behaviors for COVID-19. For information sources, reliable sources, such as the government, were reported to be associated with a higher probability of adhering to preventive behaviors [11,23]. For media, TV news and news websites have also been reported to influence compliance with preventive behaviors [16,18]. In contrast, the association between social media and compliance with preventive behaviors differed according to the type of social media used in previous studies [10,21,22]. We investigated the association between information source usage and preventive behavior by employing more detailed categories of information sources, especially social media. Our results add to the knowledge that even on SNS, the direction of association with compliance with preventive behavior differed by the type of information source (e.g., Twitter was positively and Instagram and Facebook were negatively associated with preventive behavior). In addition, the association differed according to the type of preventive behavior.

A previous review suggested that exposure to the media directly changes individual health behavior through (1) changes in emotion and perception of the behavior, (2) lowering obstacles to change the behavior, and (3) recognizing the social norms related to the behavior [24]. In addition, as several major health behavior theories include knowledge as a fundamental factor to change health behavior, we considered that providing knowledge is also an important mechanism between exposure to information and preventive behaviors [25,26]. Previous studies also suggested that exposure to social media influenced preventive behavior by increasing negative emotions toward COVID-19; meanwhile, exposure to mass media influenced preventative behavior compliance through social norms [13,27]. Another study suggested that media exposure affects preventive behavior compliance through negative emotions and knowledge [17]. A previous study also suggested that knowledge is associated with both risk perception and preventive behavior for COVID-19 [10]. Therefore, as described in the previous review, information sources and media, including social media, can influence individual behavior by providing emotional changes, knowledge, and social norms [24].

We observed that the degree or direction of the association of compliance with preventive behaviors for COVID-19 differed according to the types of information sources. We considered that these differences were caused by the amount of beneficial information on preventive behaviors provided by each source or media. In the present study, most of the information sources were not significantly associated with all four preventive behaviors. Therefore, we postulate that if the information source provided information or messages on certain preventive behaviors, the recipients would be more likely to engage in preventive behaviors. Many researchers are worried about “misinformation” or the “infodemic” [28]. We consider that the lack of beneficial information about effective preventive measures and “information overload”, which conceal important information, may also affect public compliance with preventive behaviors for COVID-19 [29,30].

We also observed that compliance with ventilation decreased at the follow-up survey. The baseline survey was conducted in summer, and the follow-up survey was conducted in winter. Therefore, we considered that this finding could be attributed to people refraining from adhering to ventilation guidelines owing to low outside temperatures in winter. A ventilation system that can maintain room temperature would contribute to higher compliance with ventilation in winter.

In the public health viewpoint, our result suggested that “medical workers”, “professionals”, “the government”, “Twitter”, “news website”, and “TV news” were positively associated with compliance with multiple preventive behaviors. These information sources play an important role in providing beneficial information for preventing COVID-19. However, not all people received information from these information sources. Therefore, developing a strategy of increasing access to these sources or media may increase compliance for preventive behavior against COVID-19. Our descriptive results also suggest that the proportion of usage of each information source differed by sociodemographic and socioeconomic status. It may also be important to consider which information source is most effective in providing beneficial information to a specific population.

The present study has several limitations. First, our survey was based on an Internet survey, which may have led to selection bias. However, in order to mitigate this limitation, we conducted an inverse probability weighting method based on the distribution obtained from nationally representative data in order to increase the representativeness of the study sample [31]. Self-reported questionnaires are vulnerable to information bias. The self-reported responses are usually different from the actual condition; therefore, the prevalence is underestimated or overestimated. In addition, such non-differential misclassification of the actual condition leads to underestimation of the association [32]. In addition, the accuracy of the questions employed in this study has not been confirmed previously, and has also increased the degree of misclassification. Third, although we included the confounders, unknown or residual confounders could have impacted the study findings. Further analysis that is robust to unmeasured confounders and employs more precise measurements would contribute to checking the robustness of the results of the present study. Fourth, our study did not consider attitudes or willingness to engage in preventive behaviors. A previous study investigating the association of socioeconomic status with anxiety or preventive behavior for radiation exposure suggested that people with low socioeconomic status tended to have more anxiety than those with high socioeconomic status, but were less engaged in preventive behavior [33]. Based on knowledge, attitude, and behavior models, there is a gap between attitude and behavior [34]; therefore, future research that considers attitudes toward preventive behavior and obstructing factors to engage in preventive behavior is required. Fifth, we could not consider the correlations between the use of each information source. Supplementary Table S3 presents the correlations between the usage of each information source, and we observed moderate correlations between several variables (phi coefficient > 0.4). A future study considering the patterns of information usage using unsupervised machine learning techniques would elucidate the effects of combining multiple information sources. Finally, although we considered temporal changes in compliance with preventive behaviors, we could not consider temporal changes in information usage.

In contrast, our study also had some strengths. The sample size of the present result was relatively large, and it contributed to the statistical power to detect the differences in estimates in various types of information sources. In addition, the present study used a longitudinal design and could measure individual responses twice. Therefore, we could analyze the associations between temporal changes and interpersonal correlations, and it provided more valid results than cross-sectional studies.

Our research partially revealed the longitudinal effects of information usage on the preventive behavior for COVID-19. For future research on this topic, a two-year follow-up to measure the change in preventive behavior and information usage would be insightful. Repeated measurements with accurate measures would also enable us to conduct a fixed-effect model or marginal structural model, which would contribute to eliminating confounding factors. In addition, future research which evaluates the interaction or effect modification of information usage on preventive behavior by sociodemographic or socioeconomic factors would contribute to providing beneficial knowledge on public health strategies.

## 5. Conclusions

The present study showed that various sources of information sources, especially “medical workers”, “professionals”, “the government”, “Twitter”, “news website”, and “TV news”, were associated with a higher probability of compliance with multiple preventive behaviors for COVID-19. These information sources played a key role in providing beneficial information for preventing COVID-19.

## Figures and Tables

**Table 1:** The characteristics of the participants (*n* = 18,151).

Characteristics		2020		2021	
		*n*	%	*n*	%
Preventive behaviors					
Wearing a mask	Yes	15,641	86.2	16,218	89.3
	No	2510	13.8	1933	10.7
Ventilation	Yes	8513	46.9	6927	38.2
	No	9638	53.1	11,224	61.8
Social distancing	Yes	8231	45.4	8569	47.2
	No	9920	54.6	9582	52.8
Avoiding crowds	Yes	11,356	62.6	11,187	61.6
	No	6795	37.4	6694	38.4
Information sources					
Family	Yes	10,488	57.8	—	—
	No	7663	42.2	—	—
Friends	Yes	8486	46.8	—	—
	No	9665	53.2	—	—
Workplace/School	Yes	7448	41.0	—	—
	No	10,703	59.0	—	—
Medical workers	Yes	3603	19.9	—	—
	No	14,548	80.1	—	—
Celebrities	Yes	3847	21.2	—	—
	No	14,304	78.8	—	—
Professionals	Yes	6293	34.7	—	—
	No	11,858	65.3	—	—
Government	Yes	8000	44.1	—	—
	No	10,151	55.9	—	—
Academic institution	Yes	1939	10.7	—	—
	No	16,212	89.3	—	—
Video sharing site	Yes	2952	16.3	—	—
	No	15,199	83.7	—	—
LINE	Yes	3852	21.2	—	—
	No	14,299	78.8	—	—
Twitter	Yes	2775	15.3	—	—
	No	15,376	84.7	—	—
Facebook	Yes	1271	7.0	—	—
	No	16,880	93.0	—	—
Instagram	Yes	975	5.4	—	—
	No	17,176	94.6	—	—
News website	Yes	12,833	70.7	—	—
	No	5318	29.3	—	—
Newspaper	Yes	9288	51.2	—	—
	No	8863	48.8	—	—
Magazines	Yes	2202	12.1	—	—
	No	15,949	87.9	—	—
Books	Yes	1234	6.8	—	—
	No	16,917	93.2	—	—
TV news	Yes	15,336	84.5	—	—
	No	2815	15.5	—	—
TV tabloid show	Yes	12,055	66.4	—	—
	No	6096	33.6	—	—
Radio	Yes	3695	20.4	—	—
	No	14,456	79.6	—	—
Gender	Male	9306	51.3	—	—
	Female	8845	48.7	—	—
Age	20–29	1963	10.8	—	—
	30–39	2554	14.1	—	—
	40–49	3713	20.5	—	—
	50–59	3312	18.2	—	—
	60–69	3476	19.1	—	—
	70–79	3133	17.3	—	—
Income	Q1 (Lowest)	3440	19.0	—	—
	Q2	4026	22.2	—	—
	Q3	3421	18.8	—	—
	Q4 (Highest)	3747	20.6	—	—
	Not answered	3517	19.4	—	—
Education	Junior high school/High school	5208	28.7	—	—
	Vocational school/Junior college	4046	22.3	—	—
	University/Graduate-school	8897	49.0	—	—
Family structure	Living with others	14,664	80.8	—	—
	Living alone	3487	19.2	—	—
Health literacy measured by CCHL		Mean	SD		
		3.5	0.7	—	—

Note: All predictors and covariates were measured only at baseline. CCHL, Communicative and Critical Health Literacy.

**Table 2:** The compliance with preventive behaviors for COVID-19 based on the characteristics in 2020 and 2021 (*n* = 18,151).

		Wearing a Mask (Yes: %)		Ventilation (Yes: %)		Social Distancing (Yes: %)		Avoiding Crowds (Yes: %)	
		2020	2021	2020	2021	2020	2021	2020	2021
Information sources									
Family	Yes	90.3	92.5	50.5	41.7	47.9	49.4	67.1	65.4
	No	80.5	85.0	41.9	33.4	41.9	44.3	56.3	56.5
Friends	Yes	90.3	92.3	52.0	43.0	46.1	47.9	65.8	64.1
	No	82.5	86.7	42.4	33.9	44.7	46.6	59.7	59.5
Workplace/School	Yes	88.7	90.8	48.6	42.0	42.8	44.8	62.2	61.1
	No	84.4	88.3	45.7	35.5	47.1	48.9	62.9	62.0
Medical workers	Yes	90.0	91.3	56.2	47.5	55.2	55.8	70.9	69.0
	No	85.2	88.9	44.6	35.9	42.9	45.1	60.5	59.8
Celebrities	Yes	90.3	92.0	52.8	43.7	48.7	49.1	66.1	63.8
	No	85.1	88.7	45.3	36.7	44.5	46.7	61.6	61.0
Professionals	Yes	92.3	93.8	53.7	43.2	50.3	51.6	69.2	67.1
	No	82.9	87.0	43.3	35.5	42.7	44.9	59.0	58.7
Government	Yes	91.8	93.5	52.2	42.9	50.1	51.8	68.7	67.3
	No	81.7	86.1	42.7	34.4	41.6	43.6	57.8	57.2
Academic institution	Yes	87.6	89.1	54.9	47.5	54.1	54.6	67.5	66.9
	No	86.0	89.4	45.9	37.1	44.3	46.3	62.0	61.0
Video sharing site	Yes	88.1	89.4	52.3	44.3	50.3	50.6	65.6	64.3
	No	85.8	89.3	45.9	37.0	44.4	46.6	62.0	61.1
LINE	Yes	89.6	91.1	53.5	45.5	48.6	49.4	65.9	64.1
	No	85.3	88.9	45.1	36.2	44.5	46.6	61.7	61.0
Twitter	Yes	86.6	87.7	50.4	44.4	46.3	47.5	61.1	60.9
	No	86.1	89.7	46.3	37.0	45.2	47.2	62.8	61.8
Facebook	Yes	82.7	85.8	52.7	45.9	48.9	48.5	60.4	60.0
	No	86.4	89.6	46.5	37.6	45.1	47.1	62.7	61.8
Instagram	Yes	81.0	82.3	55.4	51.1	49.4	49.3	60.6	61.9
	No	86.5	89.8	46.4	37.4	45.1	47.1	62.7	61.6
News website	Yes	90.2	92.7	48.7	39.4	46.7	48.3	65.4	63.8
	No	76.5	81.4	42.7	35.2	42.1	44.7	55.8	56.5
Newspaper	Yes	90.3	92.5	49.8	38.7	48.4	50.2	65.7	64.5
	No	81.9	86.0	43.9	37.6	42.2	44.1	59.3	58.6
Magazines	Yes	88.4	89.7	53.5	46.1	52.8	53.7	65.8	64.5
	No	85.9	89.3	46.0	37.1	44.3	46.3	62.1	61.2
Books	Yes	84.6	86.4	57.1	49.8	57.3	57.9	66.5	66.1
	No	86.3	89.6	46.2	37.3	44.5	46.4	62.3	61.3
TV news	Yes	90.0	92.4	48.1	38.6	46.7	48.5	65.2	64.0
	No	65.6	73.0	40.4	35.9	37.8	40.2	48.0	48.6
TV tabloid show	Yes	90.7	92.8	48.4	38.5	47.1	48.7	65.6	64.8
	No	77.3	82.5	44.0	37.4	42.0	44.3	56.5	55.4
Radio	Yes	88.7	90.9	52.9	41.1	51.5	52.1	67.3	64.9
	No	85.5	89.0	45.4	37.4	43.8	46.0	61.4	60.8
Gender									
Male		79.7	84.8	38.2	30.9	40.9	43.5	56.3	55.6
Female		93.0	94.1	56.1	45.6	50.0	51.1	69.1	68.0
Age									
20–29		75.4	76.5	42.9	39.4	36.5	37.0	52.2	51.1
30–39		81.6	85.3	44.4	40.3	37.1	40.2	57.1	56.9
40–49		85.3	89.1	46.6	39.5	41.8	44.6	59.4	60.0
50–59		87.9	91.9	47.7	38.9	46.5	47.9	62.1	60.3
60–69		90.5	93.6	46.1	35.4	51.8	53.5	67.4	65.9
70–79		91.1	93.6	51.8	36.4	53.4	54.6	72.4	70.8
Income									
Q1 (Lowest)		84.8	87.6	47.7	36.2	45.0	46.6	62.0	61.5
Q2		88.0	91.1	46.9	38.1	45.0	47.1	63.9	62.9
Q3		84.7	88.7	44.3	36.2	41.0	44.0	60.5	59.0
Q4 (Highest)		86.0	89.1	45.1	39.1	46.2	47.1	60.3	60.4
Not answered		87.0	90.0	50.6	41.1	49.4	51.2	64.9	64.2
Education									
Junior high school/High school		86.5	89.6	47.2	36.1	44.5	46.8	61.7	61.7
Vocational school/Junior college		89.5	92.1	52.9	44.1	47.4	50.2	66.3	66.0
University/Graduate-school		84.5	88.0	44.0	36.7	45.0	46.1	61.2	59.6
Family structure									
Living with others		87.2	90.3	47.3	39.0	46.4	48.4	63.8	63.0
Living alone		81.9	85.5	45.1	34.5	41.0	42.3	57.5	56.0
Health literacy measured by CCHL (Rounded)									
1		69.6	75.9	44.8	36.3	44.4	45.9	54.1	54.1
2		81.9	83.4	45.5	37.7	39.5	42.8	58.3	55.7
3		80.8	85.2	43.2	36.3	40.8	42.9	57.3	56.6
4		91.5	93.9	49.2	38.9	48.9	50.5	66.9	66.2
5		91.9	93.2	60.5	49.1	60.0	60.4	74.5	71.8

Note: CCHL, Communicative and Critical Health Literacy.

**Table 3:** The association between each information source and preventive behavior for COVID-19 (*n* = 18,151).

Absolute Difference in % [95%CI] ^†^	Wearing a Mask		Ventilation		Social Distancing		Avoiding Crowds	
	Model 1 ^‡^	Model 2 ^§^	Model 1 ^‡^	Model 2 ^§^	Model 1 ^‡^	Model 2 ^§^	Model 1 ^‡^	Model 2 ^§^
Time (2020→2021)	1.8 [−0.6, 4.2]	1.8 [−0.6, 4.2]	−9.3 [−11.2, −7.5] ***	−9.3 [−11.2, −7.5] ***	3.0 [1.2, 4.8] **	3.0 [1.2, 4.8] **	−1.2 [−3.7, 1.2]	−1.2 [−3.7, 1.2]
People/institution								
Family	5.3 [2.5, 8.1] ***	1.9 [−1.4, 4.2]	6.0 [3.0, 9.1] ***	2.4 [−1.8, 6.6]	1.5 [−1.5, 4.4]	1.4 [−2.9, 5.6]	8.1 [5.3, 10.9] ***	6.7 [2.7, 10.7] **
Friends	5.4 [2.9, 7.9] ***	1.8 [−0.5, 4.1]	6.4 [3.1, 9.6] ***	2.1 [−2.1, 6.3]	−0.7 [−3.6, 2.2]	−3.7 [−7.7, 0.2]	4.2 [1.3, 7.0] **	−0.9 [−4.6, 2.8]
Workplace/School	6.7 [3.8, 9.6] ***	4.4 [1.7, 7.2] **	4.9 [2.0, 7.8] **	0.2 [−3.1, 3.4]	0.3 [−2.4, 2.9]	−1.7 [−4.8, 1.3]	3.7 [0.8, 6.6] *	−0.5 [−3.7, 2.7]
Medical workers	2.0 [−1.6, 5.6]	−1.0 [−3.5, 1.4]	9.6 [4.7, 14.5] ***	5.9 [1.2, 10.6] *	11.2 [7.1, 15.4] ***	10.8 [6.3, 15.2] ***	6.5 [2.2, 10.7] **	3.7 [−0.5, 7.8]
Celebrities	2.1 [−1.4, 5.7]	−2.7 [−6.2, 0.8]	7.0 [3.2, 10.9] ***	0.8 [−3.8, 5.3]	1.9 [−1.2, 5.1]	−2.2 [−6.4, 2.0]	1.6 [−1.8, 5.0]	−4.7 [−9.0, −0.3] **
Professionals	6.6 [4.6, 8.6] ***	5.7 [2.1, 9.3] **	8.6 [4.8, 12.4] ***	5.0 [0.1, 10.0] *	4.8 [1.2, 8.4] **	3.0 [−1.7, 7.7]	6.8 [3.3, 10.2] ***	4.7 [−0.1, 9.4]
Government	5.5 [2.4, 8.7] **	3.9 [1.3, 6.5] **	5.3 [2.0, 8.6] **	1.0 [−2.6, 4.5]	5.1 [1.9, 8.3] **	2.9 [−0.4, 6.3]	8.9 [5.4, 12.4] ***	6.7 [3.4, 9.9] ***
Academic institution	−1.3 [−7.8, 5.2]	−5.5 [−11.0, 0.0]	9.4 [1.9, 16.9] *	4.2 [−3.3, 11.7]	7.8 [0.2, 15.4] *	3.8 [−4.3, 11.8]	4.8 [−1.0, 10.6]	−0.3 [−6.1, 5.5]
Media								
Video sharing site	4.1 [1.8, 6.4] **	2.4 [−0.6, 4.2]	6.3 [2.3, 10.3] **	2.9 [−0.7, 6.6]	5.4 [1.6, 9.2] **	4.5 [0.7, 8.3] *	4.4 [0.4, 8.3] *	2.4 [−1.4, 6.2]
LINE	3.7 [0.7, 6.8] *	1.6 [−0.7, 4.0]	4.4 [0.6, 8.1] *	0.9 [−2.3, 4.2]	2.2 [−0.9, 5.2]	−0.2 [−2.9, 2.6]	1.9 [−1.6, 5.5]	−1.4 [−4.6, 1.8]
Twitter	4.1 [−0.6, 8.7]	3.8 [0.6, 7.0] *	7.6 [3.5, 11.6] ***	4.7 [1.0, 8.3] *	6.3 [2.8, 9.9] ***	6.4 [3.0, 9.8] ***	3.5 [−0.9, 7.9]	1.3 [−3.9, 6.5]
Facebook	−2.6 [−9.0, 3.7]	0.9 [−6.4, 8.3]	2.1 [−5.3, 9.5]	−3.3 [−10.8, 4.1]	−1.6 [−6.7, 3.5]	−6.8 [−12.2, −1.3] *	0.4 [−6.7, 7.6]	−0.3 [−8.3, 7.6]
Instagram	−10.9 [−19.7, −2.0] *	−18.9 [−28.8, −8.9] ***	2.6 [−3.0, 8.1]	−3.8 [−11.2, 3.6]	−0.5 [−5.9, 4.8]	−5.4 [−12.1, 1.3]	−0.6 [−8.6, 7.5]	−5.0 [−14.9, 4.8]
News website	11.7 [8.5, 14.8] ***	7.3 [5.1, 9.5] ***	7.2 [3.2, 11.1] ***	5.5 [1.5, 9.5] *	−0.1 [−3.3, 3.2]	−2.5 [−6.2, 1.2]	8.3 [5.0, 11.6] ***	5.5 [2.1, 8.8] **
Newspaper	6.3 [3.5, 9.1] ***	3.1 [0.9, 5.3] *	0.7 [−2.2, 3.7]	−2.8 [−5.9, −0.3] *	2.6 [−0.7, 5.9]	0.6 [−2.8, 3.9]	3.3 [−0.4, 7.0]	0.2 [−3.2, 3.6]
Magazines	0.7 [−4.3, 5.7]	−4.5 [−10.6, 1.6]	9.0 [2.1, 15.8] *	5.5 [−2.7, 13.6]	9.1 [3.6, 14.7] **	6.4 [0.1, 12.6] *	5.3 [0.1, 10.4] *	2.4 [−4.4, 9.1]
Books	1.7 [−2.1, 5.6]	4.5 [−2.4, 11.4]	8.0 [−1.2, 17.2]	1.3 [−9.0, 11.6]	9.7 [2.1, 17.4] *	5.6 [−2.2, 13.5]	3.8 [−3.9, 11.5]	0.8 [−7.9, 9.4]
TV news	17.9 [13.6, 22.3] ***	12.3 [8.2, 16.3] ***	3.3 [−0.4, 7.0]	−0.5 [−5.2, 4.2]	2.1 [−0.9, 5.1]	1.7 [−2.8, 6.1]	10.4 [6.2, 14.5] ***	5.6 [0.4, 10.7] *
TV tabloid show	9.1 [5.9, 12.4] ***	2.3 [−0.4, 5.0]	2.8 [−0.9, 6.4]	−0.3 [−4.8, 4.2]	1.3 [−2.6, 5.1]	−0.6 [−5.6, 4.3]	6.7 [3.0, 10.3] ***	2.4 [−1.9, 6.7]
Radio	3.7 [1.4, 5.9] **	1.0 [−1.4, 3.3]	9.3 [5.0, 13.6] ***	7.8 [3.7, 11.8] ***	4.3 [0.0, 8.7]	2.0 [−2.3, 6.4]	4.7 [1.0, 8.5] *	2.5 [−0.6, 5.7]

^†^ The estimates were adjusted for age, gender, income, educational attainment, family structure, and health literacy. ^‡^ Model 1: Each predictor included separately in the model. ^§^ Model 2: All predictors of people-, institution-, or media-based information sources included simultaneously with time in the model. Note: * *p* < 0.05, ** *p* < 0.01, *** *p* < 0.001.

## Data Availability

Data are available upon reasonable request.

## Supplementary Materials

The following supporting information can be downloaded at: https://www.mdpi.com/article/10.3390/healthcare10030521/s1. Supplementary Figure S1: The participants flow for analytic sample; Supplementary Table S1: The differences of baseline characteristics between original and analyzed population; Supplementary Table S2: The characteristics of participants using each information source; Supplementary Table S3: The correlation between the use of information sources.
