# Supplementary material for: Information Usage and Compliance with Preventive Behaviors for COVID-19: A Longitudinal Study with Data from the JACSIS 2020/JASTIS 2021
Source: Healthcare (Basel). 2022 Mar 13;10(3):521. doi: 10.3390/healthcare10030521 (PMC8954039; doi:10.3390/healthcare10030521)
Supplement: Supplementary file 1 [file healthcare-10-00521-s001.zip › healthcare-1591910-supplementary.pdf]

**Title: Information Usage and Compliance with Preventive Behaviors for COVID-19: A Longitudinal Study with Data from the JACSIS 2020/JASTIS 2021**

Authors: Taro Kusama, Sakura Kiuchi, Kenji Takeuchi, Takaaki Ikeda, Noriko Nakazawa, Anna Kinugawa, Ken Osaka, and Takahiro Tabuchi

## Supplementary Materials

**Contents:**

- Supplementary Figure S1. The participants flow for analytic sample (n=18,151).
- Supplementary Table S1. The differences of baseline characteristics between original and analyzed population.
- Supplementary Table S2. The characteristics of participants using each information source (n=18,151).
- Supplementary Table S3. The correlation between the use of information sources (n=18,151).

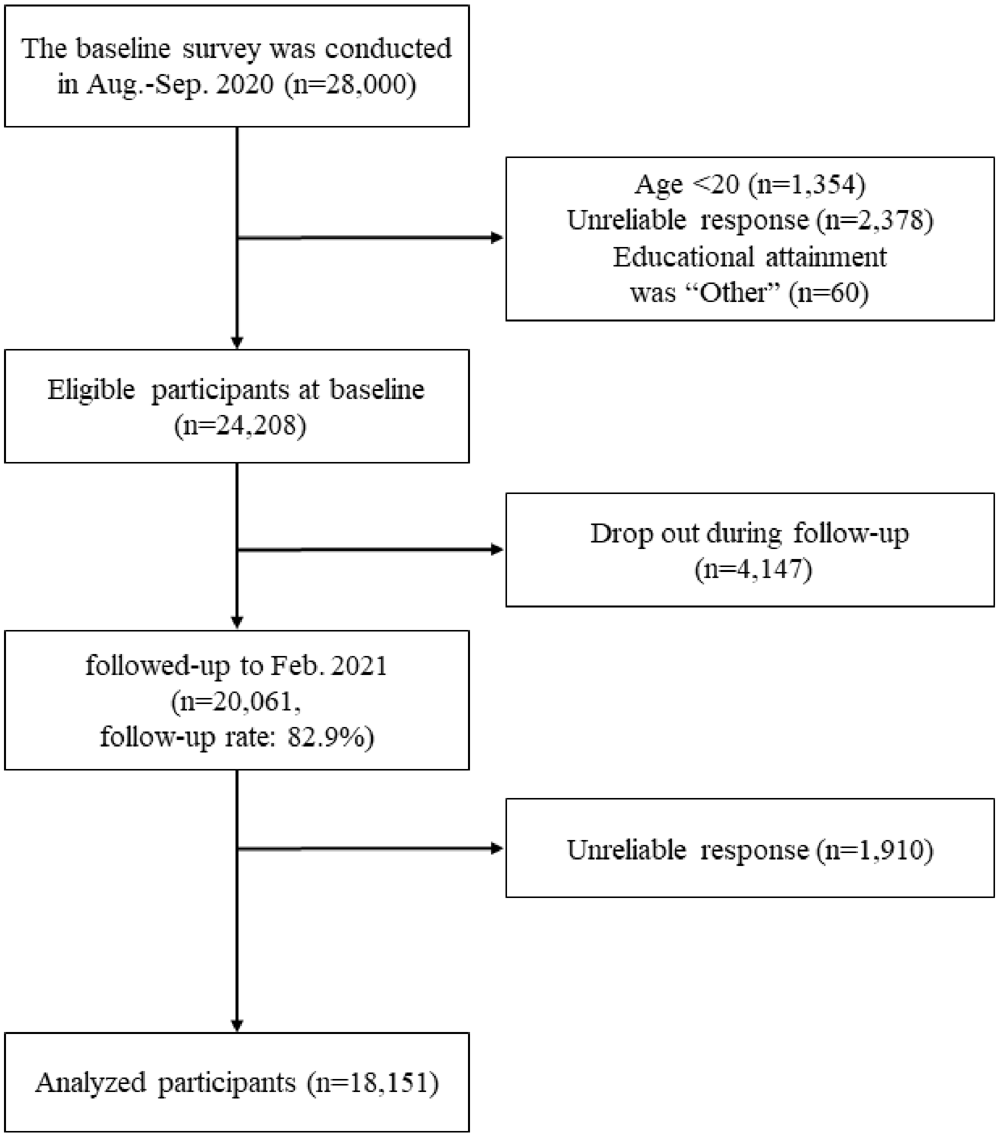


**Supplementary Figure S1. The participants flow for analytic sample (n=18,151).**

**Supplementary Table S1. The differences of baseline characteristics between original and analyzed population.**

| Characteristics at baseline | | Original (n=26,646) n | Original (n=26,646) % | Analyzed (n=18,151) n | Analyzed (n=18,151) % | Standardized difference [a] |
|---|---|---|---|---|---|---|
| **Wearing a mask** | Yes | 22,770 | 85.5 | 15,641 | 86.2 | 0.02 |
| | No | 3,876 | 14.5 | 2,510 | 13.8 | 0.02 |
| **Ventilation** | Yes | 12,639 | 47.4 | 8,513 | 46.9 | 0.01 |
| | No | 14,007 | 52.6 | 9,638 | 53.1 | 0.01 |
| **Social distancing** | Yes | 11,985 | 45.0 | 8,231 | 45.4 | 0.01 |
| | No | 14,661 | 55.0 | 9,920 | 54.6 | 0.01 |
| **Avoiding the crowds** | Yes | 16,619 | 62.4 | 11,356 | 62.6 | 0.00 |
| | No | 10,027 | 37.6 | 6,795 | 37.4 | 0.00 |
| **Family** | Yes | 15,393 | 57.8 | 10,488 | 57.8 | 0.00 |
| | No | 11,253 | 42.2 | 7,663 | 42.2 | 0.00 |
| **Friends** | Yes | 12,620 | 47.4 | 8,486 | 46.8 | 0.01 |
| | No | 14,620 | 52.6 | 9,665 | 53.2 | 0.01 |
| **Workplace/School** | Yes | 11,219 | 42.1 | 7,448 | 41.0 | 0.02 |
| | No | 15,427 | 57.9 | 10,703 | 59.0 | 0.02 |
| **Medical workers** | Yes | 5,383 | 20.2 | 3,603 | 19.9 | 0.01 |
| | No | 21,263 | 79.8 | 14,548 | 80.1 | 0.01 |
| **Celebrities** | Yes | 5,871 | 22.0 | 3,847 | 21.2 | 0.02 |
| | No | 20,775 | 78.0 | 14,304 | 78.8 | 0.02 |
| **Professionals** | Yes | 9,204 | 34.5 | 6,293 | 34.7 | 0.00 |
| | No | 17,442 | 65.5 | 11,858 | 65.3 | 0.00 |
| **Government** | Yes | 11,731 | 44.0 | 8,000 | 44.1 | 0.00 |
| | No | 14,915 | 56.0 | 10,151 | 55.9 | 0.00 |
| **Academic institution** | Yes | 2,962 | 11.1 | 1,939 | 10.7 | 0.01 |
| | No | 23,684 | 88.9 | 16,212 | 89.3 | 0.01 |
| **Video sharing site** | Yes | 4,532 | 17.0 | 2,952 | 16.3 | 0.02 |
| | No | 22,114 | 83.0 | 15,199 | 83.7 | 0.02 |
| **LINE** | Yes | 5,981 | 22.5 | 3,852 | 21.2 | 0.03 |
| | No | 20,665 | 77.5 | 14,299 | 78.8 | 0.03 |
| **Twitter** | Yes | 4,468 | 16.8 | 2,775 | 15.3 | 0.04 |
| | No | 22,178 | 83.2 | 15,376 | 84.7 | 0.04 |
| **Facebook** | Yes | 1,972 | 7.4 | 1,271 | 7.0 | 0.02 |
| | No | 24,674 | 92.6 | 16,880 | 93.0 | 0.02 |
| **Instagram** | Yes | 1,726 | 6.5 | 975 | 5.4 | 0.05 |
| | No | 24,920 | 93.5 | 17,176 | 94.6 | 0.05 |
| **News website** | Yes | 18,674 | 70.1 | 12,833 | 70.7 | 0.01 |
| | No | 7,972 | 29.9 | 5,318 | 29.3 | 0.01 |
| **Newspaper** | Yes | 12,984 | 48.7 | 9,288 | 51.2 | 0.05 |
| | No | 13,662 | 51.3 | 8,863 | 48.8 | 0.05 |
| **Magazines** | Yes | 3,234 | 12.1 | 2,202 | 12.1 | 0.00 |
| | No | 23,412 | 87.9 | 15,949 | 87.9 | 0.00 |
| **Books** | Yes | 1,902 | 7.1 | 1,234 | 6.8 | 0.01 |
| | No | 24,744 | 92.9 | 16,917 | 93.2 | 0.01 |
| **TV news** | Yes | 22,092 | 82.9 | 15,336 | 84.5 | 0.04 |
| | No | 4,554 | 17.1 | 2,815 | 15.5 | 0.04 |
| **TV tabloid show** | Yes | 17,383 | 65.2 | 12,055 | 66.4 | 0.03 |
| | No | 9,263 | 34.8 | 6,096 | 33.6 | 0.03 |
| **Radio** | Yes | 5,392 | 20.2 | 3,695 | 20.4 | 0.00 |
| | No | 21,254 | 79.8 | 14,456 | 79.6 | 0.00 |
| **Gender** | Male | 13,392 | 50.3 | 9,306 | 51.3 | 0.02 |
| | Female | 13,254 | 49.7 | 8,845 | 48.7 | 0.02 |
| **Income** | Q1 (Lowest) | 5,157 | 19.3 | 3,440 | 19.0 | 0.01 |
| | Q2 | 5,779 | 21.7 | 4,026 | 22.2 | 0.01 |
| | Q3 | 5,066 | 19.0 | 3,421 | 18.8 | 0.01 |
| | Q4 (Highest) | 5,342 | 20.1 | 3,747 | 20.6 | 0.01 |
| | Not answered | 5,302 | 19.9 | 3,517 | 19.4 | 0.01 |
| **Education** [b] | Junior high school/High school | 7,666 | 28.8 | 5,208 | 28.7 | 0.00 |
| | Vocational school/Junior college | 6,075 | 22.9 | 4,046 | 22.3 | 0.01 |
| | University/Graduate-school | 12,830 | 48.3 | 8,897 | 49.0 | 0.01 |
| **Family structure** | Living with others | 21,371 | 80.2 | 14,664 | 80.8 | 0.02 |
| | Living alone | 5,275 | 19.8 | 3,487 | 19.2 | 0.02 |
| | | **Mean** | **SD** | **Mean** | **SD** | |
| **Age** | | 50.2 | 16.3 | 51.7 | 15.9 | 0.09 |
| **Health literacy measured by CCHL** | | 3.4 | 0.7 | 3.5 | 0.7 | 0.02 |

[a] The difference in distributions of variables at baseline was evaluated by standardized difference, which indicates that if its value is <0.1, then the difference is negligible.
Ref. Austin PC. An Introduction to Propensity Score Methods for Reducing the Effects of Confounding in Observational Studies. Multivariate Behav Res 2011; 46: 399–424

[b] Those who answered their educational attainment as "the others" was excluded. The number of original populations is n=26,571

**Supplementary Table S2. The characteristics of participants using each information source (n=18,151).**

**(A) People/institution-based information sources**

| The proportion of user (%) | Family (n=10,488) | Friends (n=8,486) | Workplace /School (n=7,448) | Medical workers (n=3,603) | Celebrities (n=3,847) | Professionals (n=6,293) | Government (n=8,000) | Academic institution (n=1,939) |
|---|---|---|---|---|---|---|---|---|
| **Total** | 57.8 | 46.8 | 41.0 | 19.9 | 21.2 | 34.7 | 44.1 | 10.7 |
| **Gender** | | | | | | | | |
| Male | 53.1 | 41.8 | 44.4 | 19.9 | 17.8 | 30.2 | 41.8 | 11.8 |
| Female | 62.8 | 51.9 | 37.6 | 19.8 | 24.8 | 39.4 | 46.4 | 9.6 |
| **Age** | | | | | | | | |
| 20-29 | 59.3 | 49.6 | 53.5 | 13.5 | 22.9 | 30.3 | 39.4 | 17.8 |
| 30-39 | 61.2 | 47.8 | 53.2 | 15.9 | 20.6 | 29.6 | 39.2 | 9.2 |
| 40-49 | 57.4 | 48.4 | 54.2 | 17.8 | 23.6 | 31.9 | 43.8 | 9.8 |
| 50-59 | 52.9 | 44.6 | 49.1 | 18.0 | 22.8 | 34.3 | 46.0 | 10.1 |
| 60-69 | 57.5 | 45.8 | 30.0 | 22.4 | 19.5 | 38.0 | 46.0 | 9.2 |
| 70-79 | 59.9 | 45.6 | 11.3 | 28.7 | 17.9 | 41.5 | 47.0 | 10.8 |
| **Income** | | | | | | | | |
| Q1 (Lowest) | 51.5 | 39.7 | 28.4 | 20.1 | 19.7 | 30.4 | 39.1 | 9.0 |
| Q2 | 59.6 | 48.4 | 36.7 | 20.7 | 21.6 | 35.5 | 44.4 | 9.3 |
| Q3 | 58.8 | 49.8 | 50.8 | 18.4 | 21.5 | 35.5 | 45.7 | 10.5 |
| Q4 (Highest) | 61.7 | 51.8 | 56.7 | 21.7 | 21.8 | 37.8 | 50.3 | 14.9 |
| Not answered | 56.7 | 43.5 | 32.1 | 18.1 | 21.4 | 33.8 | 40.5 | 9.7 |
| **Education** | | | | | | | | |
| Junior high school/High school | 56.0 | 44.8 | 32.7 | 19.8 | 21.3 | 30.6 | 39.1 | 7.0 |
| Vocational school/Junior college | 58.8 | 49.7 | 40.7 | 19.7 | 24.9 | 37.4 | 43.8 | 8.4 |
| University/Graduate-school | 58.4 | 46.5 | 46.1 | 20.0 | 19.4 | 35.8 | 47.1 | 13.9 |
| **Family structure** | | | | | | | | |
| Living with others | 63.0 | 47.6 | 40.6 | 20.7 | 21.4 | 35.4 | 44.8 | 10.4 |
| Living alone | 36.0 | 43.3 | 42.7 | 16.5 | 20.3 | 31.8 | 41.1 | 11.8 |
| **Health literacy measured by CCHL (Rounded)** | | | | | | | | |
| 1 | 35.9 | 28.9 | 28.5 | 17.0 | 13.4 | 19.3 | 24.1 | 11.1 |
| 2 | 57.2 | 45.6 | 40.3 | 18.8 | 22.1 | 32.1 | 38.3 | 10.8 |
| 3 | 52.3 | 41.1 | 37.0 | 16.9 | 18.2 | 27.4 | 35.7 | 7.5 |
| 4 | 63.3 | 51.9 | 44.6 | 22.0 | 23.6 | 41.3 | 51.8 | 12.4 |
| 5 | 59.4 | 53.5 | 46.5 | 27.4 | 25.4 | 42.3 | 56.4 | 21.4 |

**(B) Media-based information sources**

| The proportion of user (%) | Video sharing site (n=2,952) | LINE (n=3,852) | Twitter (n=2,775) | Facebook (n=1,271) | Instagram (n=975) | News website (n=12,833) | Newspaper (n=9,288) | Magazines (n=2,202) | Books (n=1,234) | TV news (n=15,336) | TV tabloid show (n=12,055) | Radio (n=3,695) |
|---|---|---|---|---|---|---|---|---|---|---|---|---|
| **Total** | 16.3 | 21.2 | 15.3 | 7.0 | 5.4 | 70.7 | 51.2 | 12.1 | 6.8 | 84.5 | 66.4 | 20.4 |
| **Gender** | | | | | | | | | | | | |
| Male | 17.9 | 18.0 | 14.2 | 8.1 | 4.8 | 68.6 | 54.1 | 13.6 | 7.7 | 81.3 | 62.0 | 23.7 |
| Female | 14.6 | 24.6 | 16.5 | 5.8 | 6.0 | 72.9 | 48.1 | 10.6 | 5.9 | 87.9 | 71.0 | 16.8 |
| **Age** | | | | | | | | | | | | |
| 20-29 | 18.6 | 32.0 | 44.8 | 7.9 | 15.5 | 65.6 | 26.2 | 8.4 | 6.3 | 69.2 | 47.6 | 10.9 |
| 30-39 | 14.0 | 24.4 | 22.5 | 8.0 | 7.8 | 72.6 | 29.5 | 8.1 | 4.6 | 77.5 | 58.1 | 13.6 |
| 40-49 | 15.3 | 21.2 | 15.1 | 7.7 | 4.9 | 75.1 | 43.6 | 10.5 | 5.6 | 81.7 | 62.8 | 17.0 |
| 50-59 | 15.3 | 19.4 | 11.4 | 6.6 | 3.8 | 74.1 | 52.7 | 11.0 | 5.4 | 86.7 | 66.7 | 20.7 |
| 60-69 | 16.7 | 19.1 | 6.4 | 5.9 | 2.5 | 69.8 | 65.6 | 14.7 | 7.3 | 91.3 | 76.0 | 25.0 |
| 70-79 | 18.3 | 16.1 | 5.1 | 6.4 | 2.6 | 64.6 | 75.9 | 18.0 | 11.2 | 93.3 | 78.4 | 30.2 |
| **Income** | | | | | | | | | | | | |
| Q1 (Lowest) | 16.4 | 18.4 | 14.6 | 5.8 | 5.2 | 66.5 | 47.4 | 10.1 | 5.8 | 81.6 | 65.4 | 20.6 |
| Q2 | 17.5 | 20.6 | 13.3 | 7.1 | 4.6 | 71.8 | 55.6 | 12.4 | 6.9 | 87.2 | 70.0 | 23.2 |
| Q3 | 15.8 | 23.6 | 16.6 | 7.7 | 5.6 | 74.6 | 48.9 | 12.2 | 7.2 | 84.2 | 65.4 | 18.8 |
| Q4 (Highest) | 17.3 | 23.5 | 17.6 | 9.4 | 6.7 | 75.1 | 53.4 | 15.6 | 8.5 | 85.8 | 65.2 | 20.2 |
| Not answered | 14.1 | 20.0 | 14.5 | 5.0 | 4.8 | 65.1 | 49.6 | 10.1 | 5.5 | 83.2 | 65.6 | 18.6 |
| **Education** | | | | | | | | | | | | |
| Junior high school/High school | 15.9 | 20.4 | 14.2 | 5.5 | 4.7 | 67.2 | 50.7 | 10.0 | 5.5 | 85.1 | 70.6 | 21.4 |
| Vocational school/Junior college | 16.0 | 23.2 | 19.9 | 6.7 | 5.7 | 71.5 | 48.1 | 11.3 | 5.7 | 85.4 | 70.0 | 18.9 |
| University/Graduate-school | 16.6 | 20.1 | 15.3 | 8.0 | 5.7 | 72.4 | 52.9 | 13.8 | 8.1 | 83.7 | 62.4 | 20.4 |
| **Family structure** | | | | | | | | | | | | |
| Living with others | 15.8 | 21.1 | 14.2 | 7.0 | 5.3 | 71.0 | 55.5 | 12.7 | 7.0 | 86.6 | 68.6 | 21.0 |
| Living alone | 18.2 | 21.7 | 19.9 | 7.1 | 5.9 | 69.5 | 32.9 | 9.8 | 6.1 | 75.6 | 57.1 | 17.8 |
| **Health literacy measured by CCHL (Rounded)** | | | | | | | | | | | | |
| 1 | 14.1 | 18.2 | 19.3 | 11.5 | 11.5 | 37.4 | 26.7 | 13.7 | 11.1 | 52.6 | 41.1 | 16.7 |
| 2 | 16.0 | 22.7 | 19.6 | 8.6 | 8.8 | 63.9 | 47.2 | 11.5 | 7.5 | 81.3 | 61.7 | 19.3 |
| 3 | 13.0 | 18.7 | 13.5 | 5.7 | 4.9 | 62.7 | 45.1 | 9.4 | 5.2 | 79.5 | 61.7 | 17.7 |
| 4 | 18.4 | 22.9 | 15.2 | 7.3 | 4.8 | 78.9 | 57.6 | 14.1 | 7.5 | 90.6 | 72.4 | 22.6 |
| 5 | 25.3 | 26.3 | 25.8 | 13.0 | 8.5 | 79.2 | 54.2 | 16.8 | 12.4 | 82.6 | 62.7 | 23.9 |

**Supplementary Table S3. The correlation between the use of information sources (n=18,151).**

| Phi-coefficients [a] | 1 | 2 | 3 | 4 | 5 | 6 | 7 | 8 | 9 | 10 | 11 | 12 | 13 | 14 | 15 | 16 | 17 | 18 | 19 | 20 |
|---|---|---|---|---|---|---|---|---|---|---|---|---|---|---|---|---|---|---|---|---|
| **1. Family** | 1.00 | | | | | | | | | | | | | | | | | | | |
| **2. Friends** | **0.53** | 1.00 | | | | | | | | | | | | | | | | | | |
| **3. Workplace/School** | 0.33 | **0.43** | 1.00 | | | | | | | | | | | | | | | | | |
| **4. Medical workers** | 0.22 | 0.23 | 0.15 | 1.00 | | | | | | | | | | | | | | | | |
| **5. Celebrities** | 0.21 | 0.25 | 0.19 | 0.20 | 1.00 | | | | | | | | | | | | | | | |
| **6. Professionals** | 0.27 | 0.29 | 0.17 | 0.24 | **0.49** | 1.00 | | | | | | | | | | | | | | |
| **7. Government** | 0.22 | 0.23 | 0.19 | 0.19 | 0.22 | 0.35 | 1.00 | | | | | | | | | | | | | |
| **8.Academic institution** | 0.11 | 0.15 | 0.14 | 0.20 | 0.19 | 0.25 | 0.29 | 1.00 | | | | | | | | | | | | |
| **9. Video sharing site** | 0.12 | 0.16 | 0.10 | 0.16 | 0.20 | 0.17 | 0.22 | 0.28 | 1.00 | | | | | | | | | | | |
| **10. LINE** | 0.21 | 0.26 | 0.21 | 0.15 | 0.26 | 0.20 | 0.22 | 0.18 | 0.27 | 1.00 | | | | | | | | | | |
| **11. Twitter** | 0.11 | 0.12 | 0.14 | 0.06 | 0.16 | 0.12 | 0.13 | 0.19 | 0.22 | 0.25 | 1.00 | | | | | | | | | |
| **12. Facebook** | 0.08 | 0.13 | 0.12 | 0.15 | 0.15 | 0.13 | 0.14 | 0.20 | 0.23 | 0.25 | 0.31 | 1.00 | | | | | | | | |
| **13. Instagram** | 0.11 | 0.14 | 0.13 | 0.14 | 0.19 | 0.12 | 0.10 | 0.18 | 0.24 | 0.28 | 0.37 | 0.45 | 1.00 | | | | | | | |
| **14. News website** | 0.20 | 0.20 | 0.18 | 0.08 | 0.17 | 0.20 | 0.26 | 0.09 | 0.18 | 0.18 | 0.15 | 0.11 | 0.10 | 1.00 | | | | | | |
| **15. Newspaper** | 0.16 | 0.14 | 0.04 | 0.15 | 0.10 | 0.19 | 0.18 | 0.09 | 0.08 | 0.06 | -0.06 | 0.07 | 0.03 | 0.13 | 1.00 | | | | | |
| **16. Magazines** | 0.14 | 0.18 | 0.10 | 0.20 | 0.19 | 0.20 | 0.17 | 0.21 | 0.22 | 0.19 | 0.11 | 0.20 | 0.20 | 0.12 | 0.29 | 1.00 | | | | |
| **17. Books** | 0.11 | 0.13 | 0.08 | 0.20 | 0.16 | 0.17 | 0.16 | 0.27 | 0.22 | 0.17 | 0.14 | 0.23 | 0.24 | 0.07 | 0.20 | **0.55** | 1.00 | | | |
| **18. TV news** | 0.23 | 0.19 | 0.12 | 0.09 | 0.13 | 0.19 | 0.18 | 0.02 | 0.04 | 0.12 | -0.01 | 0.03 | 0.02 | 0.32 | 0.31 | 0.11 | 0.05 | 1.00 | | |
| **19. TV tabloid show** | 0.22 | 0.20 | 0.08 | 0.11 | 0.21 | 0.23 | 0.15 | 0.02 | 0.09 | 0.15 | -0.02 | 0.06 | 0.06 | 0.24 | 0.23 | 0.15 | 0.08 | **0.55** | 1.00 | |
| **20. Radio** | 0.11 | 0.13 | 0.07 | 0.15 | 0.12 | 0.14 | 0.14 | 0.12 | 0.14 | 0.11 | 0.06 | 0.13 | 0.10 | 0.10 | 0.23 | 0.25 | 0.23 | 0.13 | 0.15 | 1.00 |

[a] Bold value indicates phi coefficient >0.4.
